# TXNIP Regulates Peripheral Glucose Metabolism in Humans 

**DOI:** 10.1371/journal.pmed.0040158

**Published:** 2007-05-01

**Authors:** Hemang Parikh, Emma Carlsson, William A Chutkow, Lovisa E Johansson, Heidi Storgaard, Pernille Poulsen, Richa Saxena, Christine Ladd, P. Christian Schulze, Michael J Mazzini, Christine Bjørn Jensen, Anna Krook, Marie Björnholm, Hans Tornqvist, Juleen R Zierath, Martin Ridderstråle, David Altshuler, Richard T Lee, Allan Vaag, Leif C Groop, Vamsi K Mootha

**Affiliations:** 1 Department of Clinical Sciences, Diabetes and Endocrinology, Lund University, University Hospital Malmö, Malmö, Sweden; 2 Steno Diabetes Center, Gentofte, Denmark; 3 Cardiovascular Division, Brigham and Women's Hospital, Cambridge, Massachusetts, United States of America; 4 Broad Institute of Harvard and MIT, Cambridge, Massachusetts, United States of America; 5 Center for Human Genetic Research, Massachusetts General Hospital, Boston, Massachusetts, United States of America; 6 Department of Genetics, Harvard Medical School, Boston, Massachusetts, United States of America; 7 Department of Physiology and Pharmacology, Section Integrative Physiology, Karolinska Institute, Stockholm, Sweden; 8 Department of Molecular Medicine and Surgical Sciences, Section Integrative Physiology, Karolinska Institutet, Stockholm, Sweden; 9 Diabetes Biology, Novo Nordisk A/S, Maaloev, Denmark; 10 Program in Molecular Medicine, Helsinki University, Helsinki, Finland; 11 Department of Systems Biology, Harvard Medical School, Boston, Massachusetts, United States of America; Yale Medical School, United States of America

## Abstract

**Background:**

Type 2 diabetes mellitus (T2DM) is characterized by defects in insulin secretion and action. Impaired glucose uptake in skeletal muscle is believed to be one of the earliest features in the natural history of T2DM, although underlying mechanisms remain obscure.

**Methods and Findings:**

We combined human insulin/glucose clamp physiological studies with genome-wide expression profiling to identify *thioredoxin interacting protein (TXNIP)* as a gene whose expression is powerfully suppressed by insulin yet stimulated by glucose. In healthy individuals, its expression was inversely correlated to total body measures of glucose uptake. Forced expression of *TXNIP* in cultured adipocytes significantly reduced glucose uptake, while silencing with RNA interference in adipocytes and in skeletal muscle enhanced glucose uptake, confirming that the gene product is also a regulator of glucose uptake. *TXNIP* expression is consistently elevated in the muscle of prediabetics and diabetics, although in a panel of 4,450 Scandinavian individuals, we found no evidence for association between common genetic variation in the *TXNIP* gene and T2DM.

**Conclusions:**

TXNIP regulates both insulin-dependent and insulin-independent pathways of glucose uptake in human skeletal muscle. Combined with recent studies that have implicated TXNIP in pancreatic β-cell glucose toxicity, our data suggest that TXNIP might play a key role in defective glucose homeostasis preceding overt T2DM.

## Abstract 2

Vamsi Mootha, Leif Groop, and colleagues report that TXNIP regulates insulin-dependent and -independent pathways of glucose uptake in human skeletal muscle and that its expression is elevated in individuals with prediabetes and type 2 diabetes.

## Editors' Summary

**Background.:**

An epidemic of diabetes mellitus is threatening world health. 246 million people (6% of the world's population) already have diabetes and it is estimated that within 20 years, 380 million people will have this chronic disease, most of them in developing countries. Diabetes is characterized by high blood sugar (glucose) levels. It arises when the pancreas does not make enough insulin (type 1 diabetes) or when the body responds poorly to insulin (type 2 diabetes). Insulin, which is released in response to high blood glucose levels, instructs muscle, fat, and liver cells to take glucose (a product of food digestion) out of the bloodstream; cells use glucose as a fuel. Type 2 diabetes, which accounts for 90% of all cases of diabetes, is characterized by impaired glucose uptake by target tissues in response to insulin (this “insulin resistance” is one of the first signs of type 2 diabetes) and inappropriate glucose release from liver cells. Over time, the pancreas may also make less insulin. These changes result in poor glucose homeostasis (inadequate control of blood sugar levels), which can cause life-threatening complications such as kidney failure and heart attacks.

**Why Was This Study Done?:**

If the world diabetes epidemic is to be halted, researchers need a better understanding of glucose homeostasis and need to identify which parts of this complex control system go awry in type 2 diabetes. This information might suggest ways to prevent type 2 diabetes developing in the first place and might reveal targets for drugs that could slow or reverse the disease process. In this study, the researchers have used multiple approaches to identify a new mediator of glucose homeostasis and to investigate whether this mediator is causally involved in the development of type 2 diabetes.

**What Did the Researchers Do and Find?:**

The researchers took small muscle samples from people who did not have diabetes before and after increasing their blood insulin levels and used a technique called “microarray expression profiling” to identify genes whose expression was induced or suppressed by insulin. One of the latter genes was *thioredoxin interacting protein (TXNIP),* a gene whose expression is strongly induced by glucose yet suppressed by insulin. They next used previously published microarray expression data to show that *TXNIP* expression was consistently higher in the muscles of patients with diabetes or prediabetes (a condition in which blood glucose levels are slightly raised) than in normal individuals. The researchers then examined whether *TXNIP* expression was correlated with glucose uptake, again using previously published data. In people with no diabetes and those with prediabetes, as glucose uptake rates increased, *TXNIP* expression decreased but this inverse correlation was missing in people with diabetes. Finally, by manipulating *TXNIP* expression levels in insulin-responsive cells grown in the laboratory, the researchers found that *TXNIP* overexpression reduced basal and insulin-stimulated glucose uptake but that reduced *TXNIP* expression had the opposite effect.

**What Do These Findings Mean?:**

These results provide strong evidence that *TXNIP* is a regulator of glucose homeostasis in people. Specifically, the researchers propose that *TXNIP* regulates glucose uptake in the periphery of the human body by acting as a glucose- and insulin-sensitive switch. They also suggest how it might be involved in the development of type 2 diabetes. Early in the disease process, a small insulin deficiency or slightly raised blood sugar levels would increase *TXNIP* expression in muscles and suppress glucose uptake by these cells. Initially, the pancreas would compensate for this by producing more insulin, but this compensation would eventually fail, allowing blood sugar levels to rise sufficiently to increase *TXNIP* expression in the pancreas. Previously published results suggest that this would induce the loss of insulin-producing cells in the pancreas, thus further reducing insulin production and glucose uptake in the periphery and, ultimately, resulting in type 2 diabetes. Although there are many unanswered questions about the exact role of *TXNIP* in glucose homeostasis, these results help to explain many of the changes in glucose control that occur early in the development of diabetes. Furthermore, they suggest that interventions designed to modulate the activity of *TXNIP* might break the vicious cycle that eventually leads to type 2 diabetes.

**Additional Information.:**

Please access these Web sites via the online version of this summary at http://dx.doi.org/10.1371/journal.pmed.0040158.

- The MedlinePlus encyclopedia has pages on diabetes
- The US National Institute of Diabetes and Digestive and Kidney Diseases has information for patients on diabetes
- Information on diabetes is available for patients and professionals from the US Centers for Disease Control and Prevention
- The American Diabetes Association provides information on diabetes for patients
- International Diabetes Federation has information on diabetes and a recent press release on the global diabetes epidemic

## Introduction

Type 2 diabetes mellitus (T2DM) is a growing worldwide epidemic that is characterized by defects in insulin secretion, failure to suppress hepatic glucose output, and impaired glucose uptake in target tissues, such as skeletal muscle and fat [1]. Impaired glucose uptake in muscle (both insulin-dependent and -independent) is believed to be one of the earliest features in the natural history of T2DM, and insulin resistance predicts future T2DM [2–4]. Although obesity is the most important risk factor for insulin resistance in the prediabetic state [5], other factors such as the toxic effects of elevated glucose (glucotoxicity) and lipids (lipotoxicity) also contribute to insulin resistance with progression of the disease [6]. The mechanisms by which glucose and lipids induce insulin resistance are not entirely clear but are thought to involve increased serine phosphorylation and deactivation of the insulin receptor [7].

In the current study, we combined evidence from human physiology, genome-wide expression profiling, human genetics, and in vitro studies to study novel mechanisms of skeletal muscle insulin resistance and to identify mediators of glucose homeostasis that may be causally involved in the development of T2DM.

## Methods

### Clinical Participants and Evaluation

Results from three separate clinical studies (studies A, B, and C) are reported here. Participants in all three studies underwent an oral glucose (75 g) tolerance test, and glucose tolerance was diagnosed according to the World Health Organization criteria [8]. Studies A and B included six nondiabetic volunteers, while study C included 96 young (aged 25–32 y) nondiabetic twins. Details of study C were described previously [9,10]. None of the study participants were engaged in vigorous exercise on a routine basis, and they were directed to avoid extreme physical exercise and alcohol intake for at least 2 d before the studies. The study participants were asked to fast for 10–12 h before examination days. Oral and written approval was obtained from each individual in all of the clinical studies. Clinical and biochemical characteristics of participants in studies A, B, and C are provided in Table S1. All clinical studies were approved by the regional Ethics Committees and were conducted according to the principles of the Helsinki Declaration.

### Clamp Studies

#### Studies A and C.

The clamp protocol for studies A and C (Figure S1) involved a 2-h hyperinsulinemic euglycemic clamp preceded by a 30-min intravenous glucose tolerance test (IVGTT) to determine first-phase insulin secretion as previously described [10]. A primed continuous infusion of a 3-[^3^H]-glucose tracer was supplied from the time point −160 min and throughout the clamp experiments. The basal glucose turnover rates (*R*_a_ and *R*_d_) were determined during steady state conditions from −70 to −40 min. After the IVGTT a primed continuous insulin infusion (40 mU/m^2^/min) was initiated and continued for 2 h. The insulin-stimulated steady state period was defined as the last 30 min of the 2-h clamp period. A variable infusion of glucose (180 g/l) enriched with tritiated glucose (100 μCi/500 ml) maintained euglycemia during insulin infusion, with monitoring of plasma glucose concentration every 5–10 min during the basal and clamp periods using an automated glucose oxidation method (Glucose Analyzer 2, Beckman Instruments, http://www.beckmancoulter.com). Indirect calorimetry was performed during the basal and insulin-stimulated steady state periods using a computerized flow-through canopy gas analyzer system (Deltarac, Datex, http://www.us.datex-ohmeda.com).

#### Study B.

The clamp protocol for study B (Figure S1) involved a 3-h hyperinsulinemic (infusion rate 40 mU/m^2^/min) euglycemic clamp in order to increase the effects of insulin on gene expression. A variable infusion of glucose (180 g/l) was used to maintain euglycemia during insulin infusion with monitoring of plasma glucose concentration every 5–10 min using an automated glucose oxidation method (Glucose Analyzer 2, Beckman Instruments). No IVGTT was performed prior to the insulin infusion. Furthermore, no glucose tracers or indirect calorimetry was performed.

### Skeletal Muscle Biopsies

From −40 to −30 min (i.e., prior to the IVGTT), a basal muscle biopsy was taken in studies A and C (Figure S1). In study B, the basal muscle biopsy was obtained before insulin stimulation (Figure S1). The second (insulin-stimulated) muscle biopsy was taken at the time point +120 min in studies A and C, whereas in study B it was taken at the time point +180 min. The muscle biopsies were obtained from the vastus lateralis muscle under local anesthesia in participants participating in all three studies using a modified Bergström needle (including suction) before and after the hyperinsulinemic euglycemic clamps. Thus, both biopsies were determined during carefully standardized basal and insulin-stimulated euglycemic conditions. Biopsies were immediately frozen in liquid nitrogen and stored at −80 °C for later analysis.

### Human Biochemical Calculations

Plasma insulin concentrations were analyzed as previously described [10]. Insulin-stimulated glucose uptake was defined as the glucose infusion rate during steady state of the clamp. The glucose uptake and glucose oxidation were expressed per kilogram of lean body mass as determined by DEXA scan as previously described [10]. Nonoxidative glucose metabolism was calculated as glucose uptake minus glucose oxidation as determined by indirect calorimetry.

### RNA Isolation, Target Preparation, and Hybridization

RNA from skeletal muscle biopsies for studies A and B were extracted by the guadinium thiocyanate method [11]. For study A, targets from human skeletal muscle biopsies [12] were hybridized to the Affymetrix HG-U133A GeneChip (http://www.affymetrix.com), whereas for study B, targets were hybridized to the Affymetrix Hu6800 GeneChip. For both studies, we considered scans only for which there were more than 10% present-calls and the GAPDH 3′/GAPDH 5′ expression ratio was below 3.

### Real-Time PCR

Extraction of total RNA from the muscle biopsies was performed with the TRI reagent (Sigma-Aldrich, http://www.sigmaaldrich.com). In addition, RNA from cultured adipocytes was extracted using RNeasy Mini kit (Qiagen, http://www1.qiagen.com). cDNA was synthesized using Superscript II RNase H-Reverse Transcriptase (muscle biopsies; Life Technologies, http://www.invitrogen.com) or RevertAid H-M-MuLV Reverse Transcriptase (cultured adipocytes; Fermentas, http://www.fermentas.com) and random hexamer primers (Life Technologies). Real-time PCR was performed in 10 μl using the ABI PRISM 7900 Sequence Detection system (Applied Biosystems, http://www.appliedbiosystems.com) according to the manufacturer's instructions. Primers and probes for *TXNIP, G0S2,* and *BCL6* mRNA were ordered as a ready-to-use mix of primers and FAM labeled probes (Applied Biosystems). *Peptidylprolyl isomerase A (cyclophilin A) (PPIA)* was used as an endogenous control to standardize the amount of cDNA added to the reactions using a ready to use mix of primers and a VIC-labeled probe (Applied Biosystems). Analysis of the expression of *cyclophilin A* on the microarrays from studies A and B suggested that its expression is not significantly altered by insulin (unpublished data). All samples were run in duplicate, and data were calculated using the standard curve method and expressed as a ratio to the cyclophilin A reference. Quantifications of *TXNIP, BCL6,* and *G0S2* mRNA levels in study C were performed in 96 participants (study C, Table S1).

### Human Adipocyte Cell Culture

Preadipocytes of human origin were obtained and passaged as previously described [13]. Fully differentiated cells were treated with media (2% human serum albumin added) supplemented with 1, 5, or 25 mM glucose with and without 1 nM insulin (a physiological concentration). After 4 h the cells were harvested for RNA extraction.

### Glucose Uptake Assays in Mouse Adipocytes and Primary Human Skeletal Muscle Cells

#### Cell culture.

Primary human satellite cells were isolated from muscle biopsies obtained from healthy human volunteers by trypsin digestion and were grown to confluent myoblasts and differentiated into myotubes as previously described in detail [14]. 3T3-L1 mouse fibroblasts (ATCC) were cultured in DMEM and differentiated by standard means [15], via media supplemented with 5 μg/ml insulin, 0.25 μM dexamethasone, and 0.5 mM 3-isobutyl-1-methylxanthine (IBMX). Uptake assays were typically performed by day 10–12 postdifferentiation for adipocytes and day 5 postdifferentiation for human myotubes.

#### RNA interference.

Human skeletal muscle cells were seeded in six well plates at 2–3 × 10^4^ cells/cm^2^. At 70%–80% confluence, myoblasts were induced to differentiate. On day 2 of differentiation, cells were transfected as described previously [16] with short interfering RNA (siRNA) directed against *TXNIP* (Dharmacon, http://www.dharmacon.com). Adipocytes were differentiated in 12-well plates, and cells were transfected with mouse *Txnip*-specific siRNA species (individual siRNA sequences, Ambion, http://www.ambion.com). We transfected 20 ng of siRNA or negative control scrambled siRNA (Ambion) in adipocytes on day 10 postdifferentiation using Express-si delivery reagent (Genospectra, http://www.panomics.com). Glucose uptake and Western analysis followed at 48–72 h posttransfection in the presence of high-glucose (25 mM) DMEM media.

#### Viral transduction.

Human *TXNIP* was subcloned into the pCDH1-MCS1-EF1-Puro vector (System Biosciences, http://www.systembio.com). *TXNIP* or an empty vector was transfected into 293TN cells (ATCC) with Fugene 6 (Roche, http://www.roche.com) to generate pseudoviral particles. GFP was cotransfected with a 1:100 ratio (pSIH1-H1-copGFP, System Biosciences), and multiplicity of infection (MOI) was determined by the number of GFP-positive cells detected by fluorescence microscopy at 96 h. Cells were cultured in low glucose (5.6 mM), transduced with an MOI of 1–2, and allowed to express viral proteins for 96 h before initiation of glucose uptake studies. Western blot analysis was performed on total cellular lysates separated on SDS-PAGE and transferred to PVDF membranes. Monoclonal antibodies were raised against full-length TXNIP as described previously [17], and antibodies to beta-actin were purchased from Sigma.

#### Glucose uptake assays.

Virally infected or siRNA gene-silenced adipocytes were serum starved for 4 h, then incubated in Krebs-Ringer-Hepes with or without 100 nM recombinant insulin for 15 min at 37 °C. We added 0.5 mCi/ml of 2-deoxy-D-[^3^H]-glucose and 0.1 mM cold 2-deoxy- D-glucose, and the cells were incubated for five minutes at 25 °C. Uptake was terminated with ice-cold PBS containing 25 mM glucose, and cells were lysed in 0.5 ml of 1% Triton X-100/0.05% SDS at 60 °C for 10 min. Lysate radioactivity was measured by scintillation counting, and the data were expressed in pmol/mg lysate protein-min. Nonspecific glucose uptake was determined by the addition of cytochalasin B to 10 μM and was subtracted from measured values. Skeletal muscle glucose uptake assays were performed essentially as above, but with 6 nM insulin stimulation, as previously described [14].

### Resequencing of *TXNIP*

We resequenced the *TXNIP* gene along with 2,200 bp upstream of the 5′ end and 1,000 bp downstream of the 3′ end in 48 individuals, 23 of whom showed expression of *TXNIP* in muscle in the highest quartile, while 25 had *TXNIP* expression in lowest quartile from our previous studies [9,18]. Genomic DNA was isolated from peripheral blood using standard procedures. We used specific primers (Applied Biosystems) to amplify the region by PCR. Sequencing was performed using Big Dye version 3.1 Cycle Sequencing kit (Applied Biosystems) according to the manufacturer's instructions, and separation was done on the ABI3730 DNA Analyzer. Each base was called using the PHRED software package [19,20], and quality scores were assigned. Sequence assembly and analysis were done using the Staden software package [21]. Three novel single nucleotide polymorphisms (SNPs), one novel insertion, and one novel deletion were identified by resequencing *TXNIP* (Table S2).

### Genetic Association Studies

We determined the pattern of genetic variation in individuals of European origin around the *TXNIP* locus (including 20 kb upstream and 10 kb downstream) using the HAPMAP database (http://www.hapmap.org) and by genotyping an additional 33 SNPs (which also include three novel SNPs identified by resequencing) in the HAPMAP panel (90 normal individuals of European descent). In total, the pattern of linkage disequilibrium around *TXNIP* was ascertained from 15 polymorphic SNPs (32 SNPs were monomorphic, and six SNP assays failed). The gene lies in a region of high linkage disequilibrium; however, most SNPs identified were rare (<10% minor allele frequency). We selected nine tag SNPs to capture all genotypes and haplotypes with *r* ^2^ over 0.8 [22]. To study the association between *TXNIP* variants and T2DM, nine tag SNPs and two missense SNPs were genotyped in ∼4,450 individuals. The sample population consisted of 333 Scandinavian parent–offspring trios, 1,189 discordant Scandinavian sib-pairs, and 1,084 case-control pairs (969 from Scandinavia and 125 from Canada). All case-control pairs were individually matched for sex, age, region of origin, and body mass index (BMI) as previously described [23]. Genotyping was performed by primer extension of multiplex products with detection by MALDI-TOF mass spectroscopy using a Sequenom platform. This study has >99% power to reject the null hypothesis of no association at *p* < 0.05 for a 10% allele assuming an OR of 1.3 and prevalence of diabetes of 8% [24]. Association of each tag SNP to type 2 diabetes was tested for each subsample, and the results were combined by Mantel-Haenszel meta-analysis. The two missense SNPs (rs6674773 and rs11537986) were monomorphic in the diabetes sample. For a subset of nondiabetic individuals in this study, fasting glucose and fasting insulin measurements were available. We tested for correlation of *TXNIP* SNP genotypes with four related quantitative phenotypes: fasting glucose, fasting insulin, homeostasis model assessment (HOMA-IR) as a measure of insulin resistance, and HOMA-β (β-cell function) as a measure of insulin secretion. For each SNP, we tested association to each quantitative trait under a general model, a dominant model, and a recessive model.

### Statistical Analysis

#### Analysis of microarray data.

All scans were subjected to global scaling to take into account intensity-related biases. For each scan, we scaled the expression intensity of all genes such that mean intensity of each scan equals mean intensity of one high-quality scan (scan GSM172123 AI for study A; scan GSM172162 for study B). We included only those probe sets for which percentage present calls were greater than 50% of all scans to remove probe sets that were not reliably detected [25]. For study A, we therefore considered a total of 5,059 out of 22,283 probe sets on HG-U133A chip. For study B, a total 1,177 out of 7,129 probe sets passed this filtering criterion.

We applied the significance analysis of microarrays [26] method for paired data by comparing pre- versus post-clamp log (base 2) transformed data (with >1.5-fold change, delta = 0.37, and median false discovery rate = 0%) to identify insulin-regulated genes from study A. Based on this analysis, we identified two insulin-induced genes, *G0S2* (2.65-fold, *p* = 0.04), and *HES1* (2.26-fold, *p* = 0.04), and three insulin-suppressed genes, *TXNIP* (2.39-fold, *p* = 0.04), *BCL6* (2.13-fold, *p* = 0.04), and *SLC19A2* (1.76-fold, *p* = 0.04) from study A, where *p*-values refer to Wilcoxon signed rank tests. Of these five genes, three *(G0S2, TXNIP,* and *BCL6)* proved to replicate in study B again using the Wilcoxon signed rank test (Table 1).

**Table 1:**
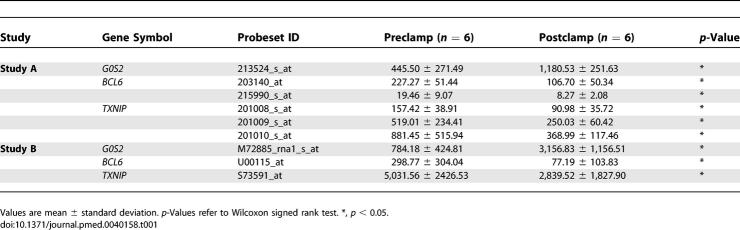
Expression of Three Insulin-Regulated Genes in Human Skeletal Muscle

#### Analysis of published microarray data.

We analyzed the expression of individual genes using previously published microarray data [18,27–29]. Normalized data from these studies [27–29] were downloaded from the Web site for the Diabetes Genome Anatomy Project (DGAP, http://www.diabetesgenome.org). For interclass, unpaired comparisons, we used the Mann-Whitney *U*-test, a nonparametric statistic, to identify differences in expression of individual genes. Spearman rank correlations were used to relate expression levels with metabolic variables.

#### Analysis of *TXNIP, BCL6,* and *G0S2* mRNA levels by real-time PCR.

**Figure 1:**
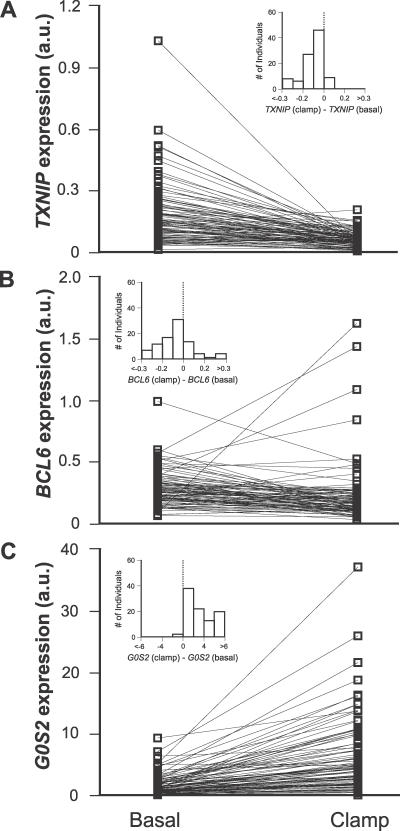
Effects of Insulin on *TXNIP, BCL6,* and *G0S2* Expression in Human Muscle In Vivo mRNA levels were measured in skeletal muscle biopsies of healthy individuals before and after the hyperinsulinemic euglycemic clamp for (A) *TXNIP* (*n* = 96, *p* < 1 × 10^−14^), (B) *BCL6* (*n* = 90, *p* < 1 × 10^−5^), and (C) *G0S2* (*n* = 95, *p* < 1 × 10^−16^). Levels were measured using real-time PCR and normalized to cyclophilin A mRNA. Inset shows the distribution of expression changes over individuals. *p*-Values refer to the Wilcoxon signed rank statistic.

The nonparametric statistic (Wilcoxon signed rank test) was used to identify significant differences between the groups for *TXNIP, BCL6,* and *G0S2* mRNA levels from study C (Figure 1).

#### Generalized estimating equation analysis.

Study C included monozygotic and dizygotic twins. To relate expression data with insulin-stimulated glucose uptake, a GEE model was used to adjust for the interdependence between twins [9,30]. In this model the correlation is assumed to be different for monozygotic and dizygotic twins. The variables included in the model were selected using a backward selection regression with a significance level set to 0.05.

#### Analysis for cellular glucose uptake studies.

Differences between groups were determined using either the paired Student t-test (adipocytes) or two-way ANOVA (myocytes). Data are presented as mean ± standard error.

## Results

### Insulin Regulates the Expression of *TXNIP, G0S2,* and *BCL6* in Human Skeletal Muscle

We began with a systematic screen to identify genes whose expression is responsive to the action of insulin in human skeletal muscle. Specifically, we obtained serial muscle biopsies from nondiabetic participants before and after a euglycemic hyperinsulinemic clamp using two different clinical protocols (study A and study B), each of which examined six individuals (see Figure S1; Table S1; and Methods). We then profiled gene expression in these biopsies using oligonucleotide microarrays. After stringent correction for multiple hypotheses testing, we identified three genes *(G0S2, TXNIP,* and *BCL6)* (Table 1) exhibiting differential expression before and after insulin treatment in both studies. All three genes were then independently validated by real-time PCR as being truly insulin responsive using archived RNA samples from a third clamp protocol (study C) that involved a panel of 96 young nondiabetic individuals (see Figure S1; Table S1; and Methods). Note that the expression of these three genes changed reproducibly across three different clamp protocols and quantitation platforms, suggesting that they represent robust changes in response to insulin.

Of these three genes, *G0S2* was strongly induced by insulin, while *TXNIP* and *BCL6* were consistently repressed by insulin (Figure 1). To our knowledge, none of these genes has previously been associated with insulin signaling. *G0S2* was recently shown to be a target gene of PPARα [31], while *BCL6* is a proto-oncogene that suppresses p53 and is implicated in the pathogenesis of human B cell lymphoma [32]. BCL6 has also been reported to interact with PPARδ [33]. TXNIP was originally identified in a yeast two-hybrid screen for proteins that bind to thioredoxin, a protein that undergoes NADPH-dependent reduction and has roles in cellular signaling and reactive oxygen species (ROS) metabolism [34]. It is notable that whereas we identify *TXNIP* expression as being strongly repressed by insulin in vivo (Figure 1), many previous studies have identified *TXNIP* as being sharply induced by glucose in a variety of cell types, including pancreatic islets [35], fibroblasts [36], mesangial kidney cells [37], rat cardiomyocytes, and vascular endothelial and smooth muscle cells [38].

### *TXNIP* Expression Is Elevated in Skeletal Muscle of Individuals with Impaired Glucose Tolerance or T2DM

**Figure 2:**
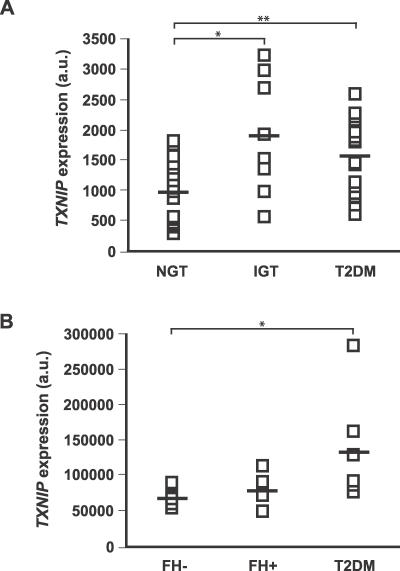
*TXNIP* Expression in Individuals with Diabetes or at Risk for Developing Diabetes (A) *TXNIP* expression levels from individuals with NGT, IGT, or T2DM, using data from our previously published microarray study [18]. * *p* < 0.02; ** *p* < 0.01, Mann-Whitney *U*-test. (B) *TXNIP* expression levels from NGT individuals with family history of T2DM (FH+) or without (FH−), as well as individuals with T2DM, using data from a previously published microarray study [27]. * *p* < 0.03, Mann-Whitney *U*-test.

Having identified three insulin-regulated genes, we sought to determine whether any of them exhibit altered expression in diabetes in humans. We analyzed two previously published microarray datasets of human diabetic and control muscle [18,27] and found that *TXNIP* expression was elevated in northern Europeans with impaired glucose tolerance (IGT) or T2DM (Figure 2A). While *TXNIP* expression was also elevated in the muscle of Mexican Americans with T2DM (Figure 2B), we found no evidence of an elevation of *TXNIP* expression in the muscle of healthy Mexican Americans with a family history of T2DM (Figure 2B). Although elevated circulating glucose levels could account for the observed differences in IGT and T2DM, it is notable that in our previous study [18], presented in Figure 2A, the muscle biopsies were obtained during conditions in which plasma glucose was clamped at the same level in all individuals.

### Suppression of *TXNIP* Expression by Insulin Requires Insulin Receptor Signaling

To determine whether the suppression of *TXNIP* by insulin in vivo is simply secondary to a decrease in circulating glucose, or due to a direct effect of insulin signaling, we examined *Txnip* gene expression using published microarray data from mice with disrupted insulin receptor signaling [28,29]. *Txnip* gene expression was more highly expressed (1.5-fold, *p* < 0.05) in the muscle of mice treated with streptozotocin, an agent that is selectively toxic to pancreatic β-cells and results in insulin deficiency and hyperglycemia. *Txnip* expression levels reverted (0.53-fold, *p* < 0.05) to baseline in the muscle of these mice following treatment with insulin, which normalizes circulating glucose levels. However, in the muscle insulin receptor knockout mouse [29], treatment of streptozotocin-treated mice with insulin failed to suppress *Txnip* expression (1.33-fold increase with insulin, *p* = 0.08), despite the fact that glucose was well controlled in these mice. These data indicate that the suppression of *Txnip* by insulin is not simply secondary to a reduction in circulating glucose concentrations, but rather or in addition, requires intact insulin receptor signaling.

### *TXNIP* Expression Is Elevated by Glucose and Suppressed by Insulin in Human Muscle and Fat Cells in Culture

**Figure 3:**
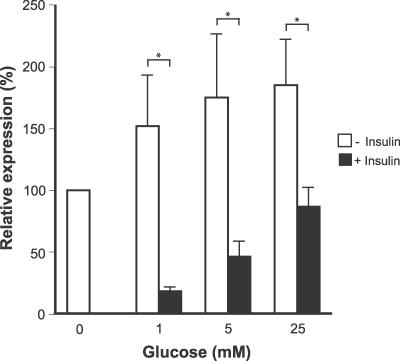
Effects of Glucose and Insulin on the Expression of *TXNIP* in Cultured Human Adipocytes Human adipocytes were cultured in the presence (black bars) or absence (white bars) of 1 nM insulin at varying levels of glucose. Data are presented as mean ± standard deviation, normalized to the untreated control. * *p* < 0.05, Mann-Whitney *U*-test.

To further confirm insulin's effect on *TXNIP* gene regulation, we analyzed *TXNIP* expression in cell culture. First, we analyzed microarray data from a published study that profiled human primary cultured myotubes following insulin treatment [39]. *TXNIP* expression was suppressed by more than 1.5-fold (*p* < 0.003) after four hours of insulin treatment, clearly demonstrating that the suppression of *TXNIP* can occur in the absence of any changes in circulating glucose concentrations. We also performed in vitro assays of *TXNIP* expression in a human adipocyte cell line (Methods) that is known to respond to insulin by stimulating glucose uptake. *TXNIP* expression increased following elevations in glucose concentration, and was suppressed by insulin (Figure 3). Together, these cell culture studies confirm that *TXNIP* expression is reciprocally regulated by insulin and glucose in human muscle and fat tissues.

### The Promoter of *TXNIP* Contains Conserved Regulatory Elements That May Account for Its Reciprocal Regulation by Insulin and Glucose

We hypothesized that the reciprocal regulation of *TXNIP* expression by insulin and glucose is likely mediated by highly conserved regulatory elements in the vicinity of its promoter (Figure S2). First, it contains tandem E-boxes that have been shown to account for the brisk response of *TXNIP* to glucose in the pancreas [40]. The promoter also contains an evolutionarily conserved binding site for the forkhead family of transcription factors. Because these forkhead family members are known to be excluded from the nucleus via Akt-mediated phosphorylation downstream of insulin signaling [41], and since *TXNIP* induction by glucose is blunted by the activation of the phosphatidylinositol-3-kinase/Akt pathway [38], this class of transcription factors represent excellent candidate mediators of the insulin-stimulated repression of *TXNIP*.

### *TXNIP* Expression Is Inversely Correlated with Insulin-Stimulated Glucose Uptake

**Figure 4:**
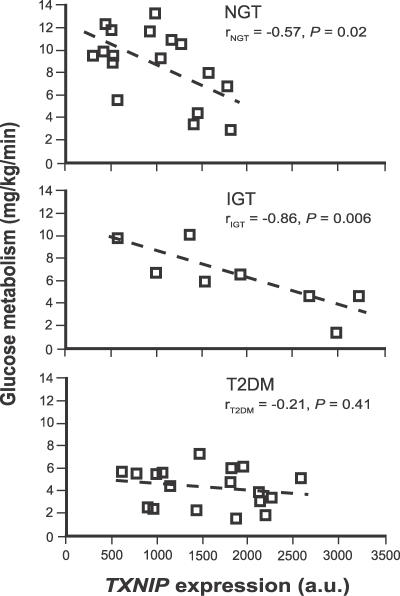
Insulin-Mediated Glucose Uptake Versus *TXNIP* Expression This relationship is shown for individuals with NGT, IGT, and T2DM. Linear regression parameters are indicated for each of the groups. Data are from our previously published microarray study [18].

We found that *TXNIP* expression is inversely correlated with the total body rate of insulin-stimulated glucose metabolism in humans (Figure 4). This relationship is robust in individuals with normal glucose tolerance (NGT) (*r* = −0.57, *p =* 0.02) or IGT (*r =* −0.86, *p =* 0.006), but not in patients with T2DM (*r =* −0.21, *p =* 0.41). To exclude the possibility that the relationship between *TXNIP* expression and glucose uptake could be a consequence, rather than a cause, of the metabolic derangements that precede T2DM we also related the expression of skeletal muscle *TXNIP* to glucose uptake in 96 healthy, young, nondiabetic individuals (Methods, study C). Since both dizygotic and monozygotic twins were included in the analysis, we used a generalized estimating equation model to correct for the relationship between twin pairs (Methods). In the analysis, we modeled insulin-stimulated glucose uptake as a function of age, sex, basal and insulin-stimulated *TXNIP* expression, zygosity, birth weight, percentage of body fat, BMI, and VO_2max_. Only four of these variables had explanatory power. Basal (*p* = 0.02) and insulin-stimulated (*p* < 0.01) *TXNIP* expression as well as BMI (*p* < 0.01) were inversely correlated, while VO_2max_ (*p* < 0.01) was positively correlated with insulin-stimulated glucose uptake (Table 2). These data are consistent with the notion that *TXNIP* is an independent determinant of insulin-stimulated glucose uptake in humans.

**Table 2:**
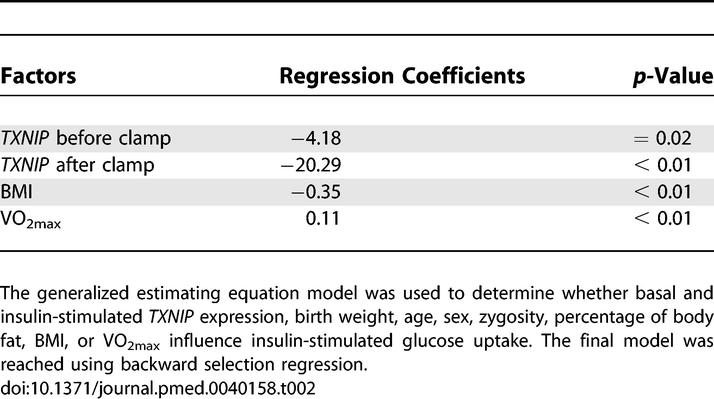
Factors Influencing Insulin-Stimulated Glucose Uptake in Humans

### *TXNIP* Expression Governs Glucose Uptake in Insulin-Responsive Cells

**Figure 5:**
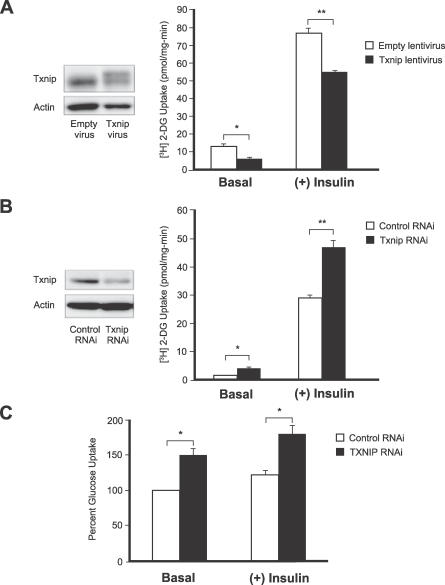
Genetic Manipulation of *TXNIP* Expression Affects Both Basal and Insulin-Stimulated Glucose Uptake in Cultured Adipocytes and in Primary Skeletal Muscle Myocytes (A) Differentiated 3T3-L1 adipocytes cultured in 5.6 mM glucose were transduced with human *TXNIP* or empty virus particles and allowed to express the genes for 96 h. Cell lysates were immunoblotted using antibodies against *TXNIP* or actin. The exogenous viral human *TXNIP* protein is present as the more slowly migrating of the two bands. Glucose uptake with and without insulin was measured as described in Methods. Each data point represents an *n* = 4. * *p* < 0.02; ** *p* < 0.005. (B) Adipocytes cultured in 25 mM glucose were transfected with siRNA against *TXNIP* or with negative control siRNA and assayed 48 h posttransfection. Immunoblotting and glucose uptake are as above. *n* = 4 for each data point. * *p* < 0.02; ** *p* < 0.001. (C) Human skeletal muscle myoblasts obtained from biopsy were differentiated into myotubes in culture and transfected with siRNA against *TXNIP* or with negative control siRNA. Glucose uptake was measured at 72 h post-transfection with *n* = 4 for each data point. Each experiment was performed at least three times. * *p* < 0.01

To directly test if *TXNIP* may regulate glucose uptake, we used genetic manipulations to alter the gene's expression in mouse and human cell lines. First we performed in vitro studies on an insulin-sensitive cell line, the adipocyte differentiated 3T3-L1. Incubation of the adipocytes with high glucose results in a potent induction of TXNIP protein (unpublished data). A 2-fold forced overexpression of *TXNIP* using lentiviral transduction resulted in both diminished basal and insulin-stimulated glucose uptake (59% and 28% reduction, respectively, Figure 5A). In agreement with this result, forced reduction of *TXNIP* expression by siRNA gene silencing achieved the opposite effect and enhanced both basal and insulin-stimulated glucose uptake (157% and 61%, respectively, Figure 5B). In addition, we examined the effects of *TXNIP* reduction in primary human skeletal muscle myocytes by siRNA gene silencing. Again, we observed a significant increase in both basal and insulin-stimulated glucose uptake (Figure 5C). Together, these in vitro studies indicate that changes in *TXNIP* expression can directly influence glucose uptake in insulin-responsive cells.

### Do Genetic Variants in the *TXNIP* Gene Increase Susceptibility to T2DM?

**Figure 6:**
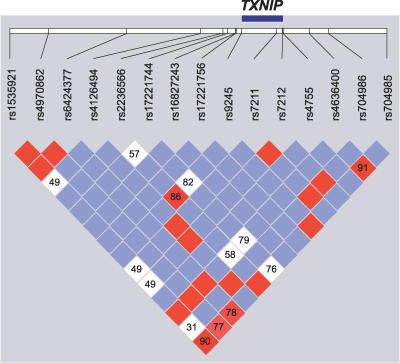
Pattern of Genetic Variation at the *TXNIP* Locus Linkage disequilibrium between SNPs was analyzed using Haploview [55], and D′ values were calculated with 95% confidence intervals. D′ plot for each square depicts the magnitude of linkage disequilibrium for a pair of markers, red indicates high D′ with high logarithm of odds (LOD) score, while white indicates low D′ with low LOD score, and blue indicates high D′ with low LOD score.

While the above studies demonstrate that TXNIP is a physiologic regulator of peripheral glucose homeostasis in humans, we were next interested in determining whether genetic variation in this gene may be associated with measures of insulin resistance or T2DM in humans. We assessed the pattern of common genetic variation encompassing the *TXNIP* gene using data from the HapMap project and by resequencing the gene (Methods; Table S2). Then we performed a genetic association study of nine “tag” SNPs that capture the most common variation observed within the vicinity of this gene (Figure 6). This study was in part motivated by the observation that *TXNIP* is located on Chromosome 1q21.1, a region that has shown perhaps the most consistent linkage to T2DM [42,43]. In a clinical sample of 4,450 Scandinavian individuals, no significant association was detected between SNPs in *TXNIP* and T2DM (Methods; Table 3). Also, no significant association was observed between the genotypes and four quantitative phenotypes: fasting glucose, fasting insulin, HOMA-IR as a measure of insulin resistance, and HOMA-β as a measure of insulin secretion (Table S3). Although we did not detect polymorphisms in *TXNIP* that are associated with T2DM, it is still formally possible that inherited variation in other genes in the *TNXIP* pathway may predispose to the risk of developing T2DM.

**Table 3:**
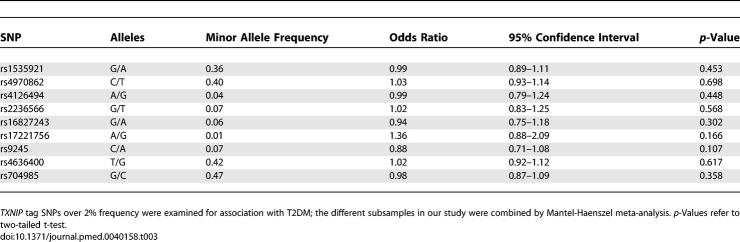
Association of *TXNIP* Tag SNPs with T2DM

## Discussion

We have combined human physiology, genome-wide expression profiling, genetic association testing, and cellular studies to spotlight TXNIP as a physiologic regulator of peripheral glucose uptake in humans. Our results build on murine studies that have established a role for this protein in the liver in the regulation of fasting:feeding transitions [44,45] and in β-cell glucose toxicity [35]. While genetic variation in *TXNIP* does not predispose one's inherited risk for developing T2DM, TXNIP nonetheless appears to play a physiologic role in glucose homeostasis in muscle and in fat. Key novel results from the current study include the finding that *TXNIP* gene expression is reciprocally regulated by insulin and by glucose, that elevations in *TXNIP* can inhibit glucose uptake, and that *TXNIP* expression levels are consistently elevated in humans with T2DM and prediabetes.

**Figure 7:**
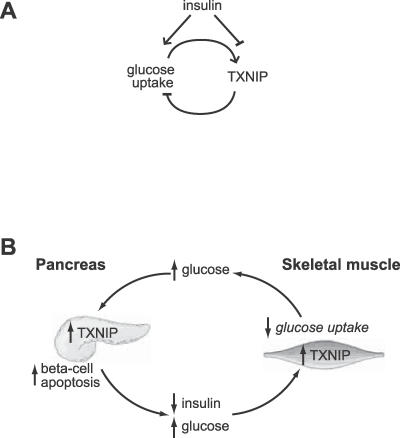
Model of *TXNIP* Regulation and Action and Its Potential Role in the Pathogenesis of T2DM (A) Previous studies have shown that glucose induces the expression of *TXNIP* in a variety of cells and tissues. In this study we show that insulin suppresses the expression of *TXNIP*. Moreover, we have found that forced expression of *TXNIP* results in reduced glucose uptake, while inhibition of *TXNIP* enhances glucose uptake. These results suggest that *TXNIP* serves as a glucose- and insulin-sensitive homeostatic switch that regulates glucose uptake in the periphery. (B) Role of *TXNIP* in glucose toxicity in the β-cell and in impaired glucose uptake in the periphery. Insulin deficiency or hyperglycemia can increase *TXNIP* levels in muscle, resulting in impaired peripheral glucose uptake. The pancreatic β-cell is initially able to compensate by secreting more insulin, but eventually the β-cell compensation fails. The resulting hyperglycemia may then elevate pancreatic β-cell *TXNIP* expression, which can induce apoptosis [40]. The loss of β-cells, in turn, results in decreased insulin production that further exacerbates peripheral IGT. The vicious cycle would eventually spiral to T2DM.

Our study shows that TXNIP is part of a negative feedback mechanism that regulates glucose uptake into cells (Figure 7A). Such a mechanism may serve to prevent excess glucose uptake or metabolism [46]. At present, it is not clear whether TXNIP influences the metabolic fate of glucose—future studies will be required to address this important issue. Because *TXNIP* expression is suppressed by insulin signaling, it appears that TXNIP influences both insulin-dependent and insulin-independent arms of glucose uptake into cells. Of note, epidemiological studies have suggested roles for both insulin-dependent and insulin-independent pathways in the development of T2DM [4].

Recent genomic studies have pointed to mitochondrial dysfunction and to ROS as possible culprits in the etiology of insulin resistance [18,27,47,48]. At present, we do not know how mitochondrial dysfunction and ROS are related to each other in the pathogenesis of insulin resistance. The current study, in combination with other recent studies, suggests that TXNIP may lie upstream of both of these processes. First, *TXNIP* expression is induced by glucocorticoids [49] and by glucose, and TXNIP elevations can stimulate ROS production [38]. Hence TXNIP represents a candidate intermediate linking diabetogenic stimuli to ROS production. Second, we have observed an inverse correlation in the expression of *TXNIP* and mitochondrial oxidative phosphorylation (unpublished data), and previous studies have shown that TXNIP can serve as a transcriptional repressor [50]. Taken together, these data raise the hypothesis that TXNIP may lie upstream of the elevations in ROS and mitochondrial dysfunction that accompany insulin resistance.

Importantly, our findings, in combination with recent studies focused on the pancreatic β-cell [40], provide molecular insights into the pathogenesis of impaired insulin secretion and action (Figure 7B) that characterize the prediabetic state [1,51]. Hyperglycemia has long been known to exacerbate β-cell failure [52,53] and impaired skeletal muscle glucose uptake [54] via a process often termed glucose toxicity. At present the molecular basis for glucose toxicity is not known, although ROS represent a candidate mediator [46,47]. A recent study [40] reported that *TXNIP* is glucose inducible in pancreatic β-cells and may mediate β-cell death through apoptosis. Our present study has led to the novel finding that *TXNIP* also plays a role in controlling peripheral glucose uptake in humans and may be an important target mediating effects of insulin. Together, these studies suggest that *TXNIP* may be involved in glucose toxicity both in β-cells and in the periphery, helping to reconcile the dynamic relationship between insulin deficiency and impaired glucose uptake that is observed in the prediabetic state (Figure 7B). Interventions aimed at modulating *TXNIP* activity may therefore help curtail this vicious cycle that eventually leads to overt T2DM.

## Supporting Information

Figure S1 Design of the Hyperinsulinemic, Euglycemic Clamp Protocols for Studies A, B, and C (352 KB PDF)

Figure S2 The Promoter of *TXNIP* Contains a Conserved Binding Site for the Tandem E-Boxes and for the Forkhead Family of Transcription Factors in Four Species (Human, Mouse, Rat, and Dog) Positions are relative to translation start site of TXNIP gene. See [56] for tandem E-boxes and [35] for forkhead family of transcription factors.
(298 KB PDF)

Table S1 Clinical and Biochemical Characteristics of Participants (58 KB DOC)

Table S2 *TXNIP* Resequencing in 48 Individuals (35 KB DOC)

Table S3 Genotype-Phenotype Correlations of TXNIP Tag SNPs with Fasting Glucose, Fasting Insulin, Insulin Resistance (Measured by HOMA-IR) and Insulin Secretion (Measured by HOMA-β Cell Function) in Nondiabetic Individuals (157 KB DOC)

### Accession Numbers

The GenBank (http://www.ncbi.nlm.nih.gov/Genbank) accession numbers for the genes are *TXNIP* (10628), *G0S2* (50486), *BCL6* (604), *HES1* (3280), *SLC19A2* (10560), *PPIA* (5478), *PPARA* (5465), and *PPARD* (5467). The microarray data have been deposited in the National Center for Biotechnology Information's Gene Expression Omnibus (GEO) database (http://www.ncbi.nlm.nih.gov/geo); series accession number is GSE7146.

## Abbreviations

BMI: body mass index
HOMA: homeostasis model assessment
IGT: impaired glucose tolerance
IVGTT: intravenous glucose tolerance test
NGT: normal glucose tolerance
ROS: reactive oxygen species
SNP: single nucleotide polymorphism
T2DM: type 2 diabetes mellitus
