# Supplementary material for: TXNIP Regulates Peripheral Glucose Metabolism in Humans
Source: PLoS Med. 2007 May 1;4(5):e158. doi: 10.1371/journal.pmed.0040158 (PMC1858708; doi:10.1371/journal.pmed.0040158)

Supplemental Figure 1

**Study A**: Six non-diabetic male volunteers

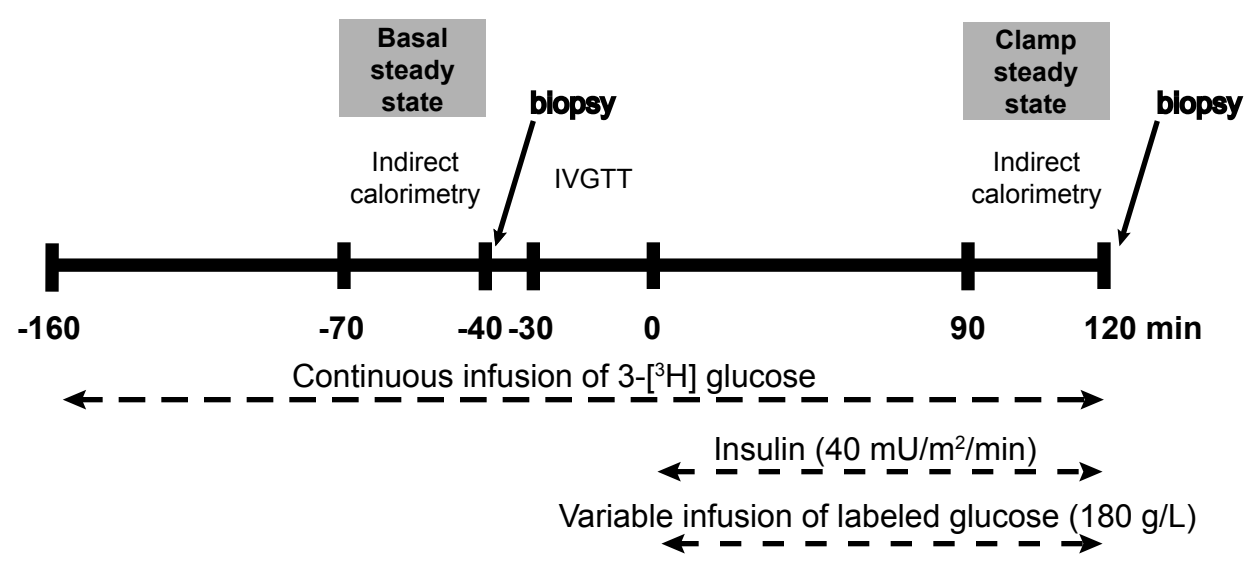


**Study B**: Six non-diabetic volunteers (M=4, F=2)

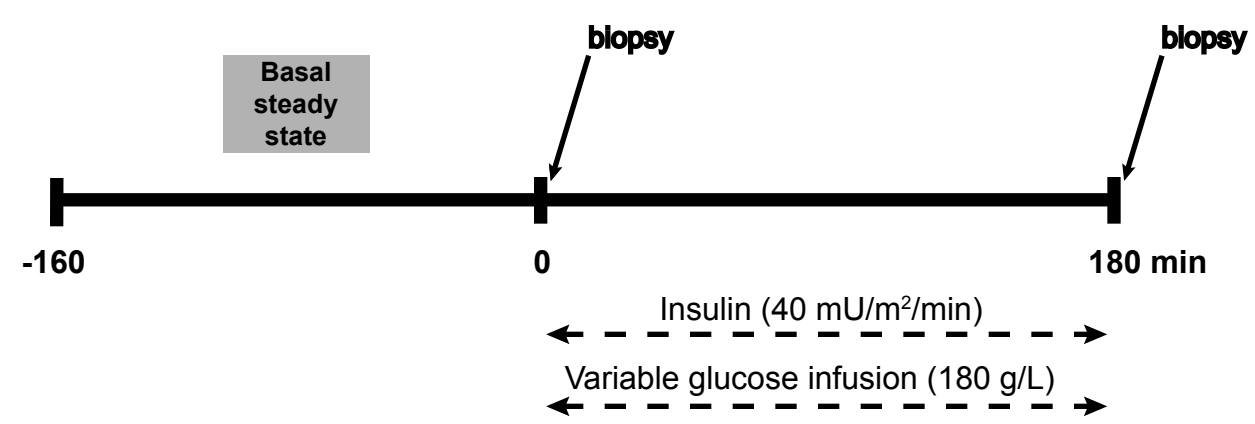


**Study C**: 96 non-diabetic volunteers (M=56, F=40)

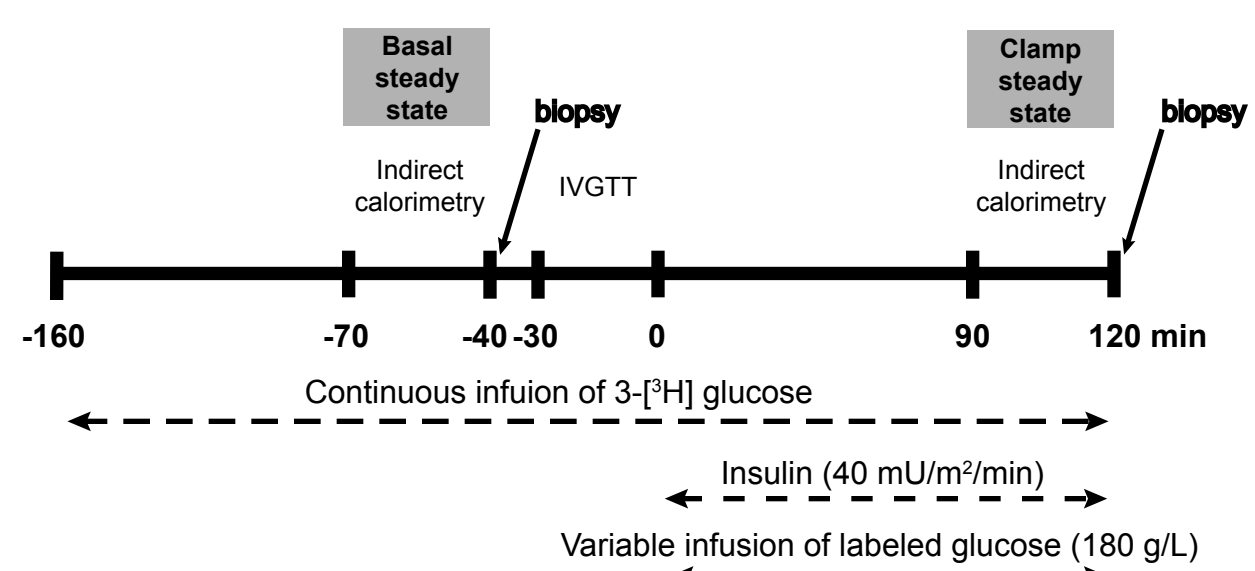

Supplement: Supplementary file 1 [file pmed.0040158.sg001.pdf]
