# Supplementary material for: TXNIP Regulates Peripheral Glucose Metabolism in Humans
Source: PLoS Med. 2007 May 1;4(5):e158. doi: 10.1371/journal.pmed.0040158 (PMC1858708; doi:10.1371/journal.pmed.0040158)
Supplement: Supplementary file 2 [file pmed.0040158.sg002.pdf]

Supplemental Figure 2

```
               FOXO                                    E-Box       E-Box
      -450                                                                       -375

human GTCGGGCTCCTGGTAAACAAGGACCGGGCAGCCAATGGGAGGGATGTGCACGAGGGCAGCACGAGCCTCCGGGCC
mouse GTTGGAGGCCTGGTAAACAAGGGCCAAGTAGCCAATGGGAGAACTGTGCACGAGGGCTGCACGAGCCTCCAGGCC
rat   GTTAGAGGCCTGGTAAACAAGGACCAAGTAGCCAATGGGAGAACTGTGCACGAGGGATGCACGAGCCTCCGGGCC
dog   GTTGGGCTCCTGGTAAACAAGGGGCGGGCAGCCAATGGGAGGGCTGTGCACGAGGGCAGCACGAGCCTCAGGGCT
      **  *   **************  *  * ************   ************  ***********  ***
```
