# Supplementary material for: TXNIP Regulates Peripheral Glucose Metabolism in Humans
Source: PLoS Med. 2007 May 1;4(5):e158. doi: 10.1371/journal.pmed.0040158 (PMC1858708; doi:10.1371/journal.pmed.0040158)
Supplement: Supplementary file 3 [file pmed.0040158.st001.doc]

**Supplemental Table 1: Clinical and biochemical characteristics of subjects**

Study A Clinical characterization of male subjects

| N | 6 |
| --- | --- |
| Age (yrs) | 55.1 ± 4.9 |
| BMI (kg/m^2^) | 31.9 ± 2.4 |
| Waist/hip ratio | 1.0 ± 0.02 |
| **OGTT p-glucose (mmol/l)** |  |
| 0 min | 6.0 ± 0.7 |
| 120 min | 7.6 ± 1.7 |
| **OGTT p-insulin (pmol/l)** |  |
| 0 min | 73.3 ± 41.6 |
| **Euglycemic hyperinsulinaemic clamp** |  |
| Basal glucose (mmol/l) | 6.2 ± 0.5 |
| Clamp glucose (mmol/l) | 4.9 ± 0.5 |
| Basal insulin (pmol/l) | 70.9 ± 87.5 |
| Clamp insulin (pmol/l) | 466.7 ± 134.8 |
| Glucose metabolism (mg/kg lbm/min) | 5.6 ± 1.6 |
| Glucose oxidation (mg/kg lbm/min) | 3.1 ± 0.8 |
| NOGM (mg/kg lbm/min) | 2.6 ± 2.0 |

**Study B** Clinical characterization of subjects

| N (M/F) | 6 (4/2) |
| --- | --- |
| Age (yrs) | 65.8 ± 6.7 |
| BMI (kg/m^2^) | 28.3 ± 4.2 |
| Waist/hip ratio | 0.9 ± 0.05 |
| **OGTT p-glucose (mmol/l)** |  |
| 0 min | 5.9 ± 0.4 |
| 120 min | 8.5 ± 1.6 |

# Study C Clinical characterization of subjects

| N (M/F, MZ/DZ) | 96 (56/40, 56/40) |
| --- | --- |
| Age (yrs) | 28 ± 1.9 |
| BMI (kg/m^2^) | 24.1 ± 3.2 |
| Waist/hip ratio | 0.8 ± 0.09 |
| **OGTT p-glucose (mmol/l)** |  |
| 0 min | 5.2 ± 0.4 |
| 120 min | 5.9 ± 0.9 |
| **OGTT p-insulin (pmol/l)** |  |
| 0 min | 34.1 ± 17.2 |
| 120 min | 138.1 ± 78.9 |
| **Euglycemic hyperinsulinaemic clamp** |  |
| Basal glucose (mmol/l) | 5.4 ± 0.4 |
| Clamp glucose (mmol/l) | 5.3 ± 0.4 |
| Basal insulin (pmol/l) | 30.7 ± 14.2 |
| Clamp insulin (pmol/l) | 401.4 ± 58.4 |
| Glucose metabolism (mg/kg lbm/min) | 11.8 ± 3.3 |
| Glucose oxidation (mg/kg lbm/min) | 4.7 ± 1.4 |
| NOGM (mg/kg lbm/min) | 7.1 ± 2.9 |

Values are mean ± SD.

**Abbreviations:** DZ, dizygotic twins; MZ, monozygotic twins; BMI, body mass index; OGTT, oral glucose tolerance test; p-, plasma; lbm, lean body mass; NOGM, non oxidative glucose metabolism.
