# Supplementary material for: TXNIP Regulates Peripheral Glucose Metabolism in Humans
Source: PLoS Med. 2007 May 1;4(5):e158. doi: 10.1371/journal.pmed.0040158 (PMC1858708; doi:10.1371/journal.pmed.0040158)
Supplement: Supplementary file 4 [file pmed.0040158.st002.doc]

**Supplemental Table 2: *TXNIP*** re-sequencing in 48 individuals

| **SNP** | **Chromosome position** | **Alleles** | **Nos. of carriers** | **Function** |
| --- | --- | --- | --- | --- |
| rs2236566 | 144148243 | G/T | 4 | locus |
| rs17221744 | 144148667 | C/T | 6 | locus |
| New insertion | 144148846 | -/A | 1 | locus |
| New SNP1 | 144148962 | T/C | 1 | locus |
| rs16827243 | 144149424 | G/A | 6 | locus |
| rs17221756 | 144149464 | A/G | 1 | locus |
| rs17215322 | 144149624 | C/G | 1 | locus |
| rs9245 | 144149938 | C/A | 6 | locus |
| New SNP2 | 144151443 | C/T | 1 | nonsynonymous |
| rs7211 | 144152977 | C/T | 3 | untranslated |
| New SNP3 | 144153389 | A/C | 2 | untranslated |
| rs7212 | 144153611 | C/G | 3 | untranslated |
| New deletion | 144153991 | A/- | 1 | untranslated |
