# Supplementary material for: TXNIP Regulates Peripheral Glucose Metabolism in Humans
Source: PLoS Med. 2007 May 1;4(5):e158. doi: 10.1371/journal.pmed.0040158 (PMC1858708; doi:10.1371/journal.pmed.0040158)
Supplement: Supplementary file 5 [file pmed.0040158.st003.doc]

**Supplemental Table 3**: **Genotype-phenotype correlations of *TXNIP* tag SNPs with fasting glucose, fasting insulin, insulin resistance**

**(measured by HOMA-IR) and insulin secretion (measured by HOMA-β** cell function) in non diabetic individuals

| **Phenotype (N)** | **N** | **Mean Trait Values (+/- SD) by genotype** | | | | | ***P* value** | | |
| --- | --- | --- | --- | --- | --- | --- | --- | --- | --- |
|  |  |  |  |  |  |  | **Neutral** | **Dom** | **Rec** |
| **rs1535921** |  | AA | AG | GG | AA/AG | AG/GG |  |  |  |
|  |  |  |  |  |  |  |  |  |  |
| Fasting p-glucose (mmoL) | 1511 | 5.8 ± 2.0 (231) | 6.1 ± 2.2 (630) | 5.9 ± 1.9 (650) | 6.0 ± 2.2 (861) | 6.0 ± 2.0 (1280) | 0.12 | 0.2 | 0.3 |
| Fasting insulin (mU/ml) | 1105 | 9.8 ± 8.2 (179) | 11.2 ± 9.7 (442) | 10.2 ± 10 (484) | 10.8 ± 9.3 (621) | 10.7 ± 9.8 (926) | 0.05 | 0.09 | 0.32 |
|  |  |  |  |  |  |  |  |  |  |
| HOMA-IR | 1097 | 2.8 ± 3.9 (178) | 3.3 ± 3.5 (438) | 2.9 ± 4.6 (481) | 3.2 ± 3.6 (616) | 3.1 ± 4.1 (919) | 0.007 | 0.05 | 0.14 |
| HOMA-β | 1097 | 100 ± 94 (178) | 107 ± 123 (438) | 96 ± 94 (481) | 105 ± 115 (616) | 101 ± 109 (919) | 0.70 | 0.40 | 0.67 |
|  |  |  |  |  |  |  |  |  |  |
| **rs4970862** |  | TT | CT | CC | TT/CT | CT/CC |  |  |  |
|  |  |  |  |  |  |  |  |  |  |
| Fasting p-glucose (mmoL) | 1667 | 5.9 ± 2.0 (320) | 6.0 ± 2.0 (766) | 5.9 ± 1.9 (581) | 6.0 ± 2.0 (1086) | 5.9 ± 1.9 (1347) | 0.76 | 0.86 | 0.43 |
| Fasting insulin (mU/ml) | 1225 | 9.9 ± 8.2 (244) | 10.5 ± 9.6 (563) | 10 ± 9.6 (418) | 10 ± 9.2 (807) | 10 ± 9.6 (981) | 0.84 | 0.86 | 0.65 |
|  |  |  |  |  |  |  |  |  |  |
| HOMA-IR | 1216 | 2.9 ± 3.6 (241) | 3.0 ± 3.9 (561) | 2.9 ± 4.0 (414) | 3.0 ± 3.8 (802) | 3.0 ± 3.9 (975) | 0.72 | 0.73 | 0.57 |
| HOMA-β | 1216 | 99 ± 93 (241) | 101 ± 109 (561) | 99 ± 102 (414) | 101 ± 105 (802) | 100 ± 106 (975) | 0.93 | 0.70 | 0.87 |
|  |  |  |  |  |  |  |  |  |  |
| **rs4126494** |  | GG | AG | AA | GG/AG | AG/AA |  |  |  |
|  |  |  |  |  |  |  |  |  |  |
| Fasting p-glucose (mmoL) | 1774 | 5.6 ± 0.5 (8) | 6.0 ± 2.1 (173) | 5.9 ± 2.0 (1593) | 6.0 ± 2.1 (181) | 5.9 ± 1.9 (1766) | 0.53 | 0.37 | 0.63 |
| Fasting insulin (mU/ml) | 1305 | 8.4 ± 1.5 (5) | 10.6 ± 10.8 (139) | 10.1 ± 9.0 (1161) | 10.5 ± 11 (144) | 10.2 ± 9.2 (1300) | 0.97 | 0.79 | 0.92 |
|  |  |  |  |  |  |  |  |  |  |
| HOMA-IR | 1296 | 2.1 ± 0.5 (5) | 3.4 ± 6 (137) | 2.9 ± 3.5 (1154) | 3.3 ± 6.0 (142) | 2.9 ± 3.8 (1291) | 0.79 | 0.52 | 0.89 |
| HOMA-β | 1296 | 92 ± 20 (5) | 92 ± 90 (137) | 99 ± 103 (1154) | 92 ± 88 (142) | 98 ± 102 (1291) | 0.70 | 0.60 | 0.57 |
|  |  |  |  |  |  |  |  |  |  |
| **rs2236566** |  | TT | GT | GG | GT/TT | GT/GG |  |  |  |
|  |  |  |  |  |  |  |  |  |  |
| Fasting p-glucose (mmoL) | 1709 | 5.5 ± 0.7 (6) | 5.7 ± 1.3 (217) | 5.9 ± 2.0 (1486) | 5.7 ± 1.9 (223) | 5.9 ± 2.0 (1703) | 0.16 | 0.06 | 0.64 |
| Fasting insulin (mU/ml) | 1283 | 10.8 ± 0.9 (2) | 8.7 ± 6.1 (163) | 10.4 ± 9.6 (1118) | 8.7 ± 9.2 (165) | 10.2 ± 9.2 (1281) | 0.11 | 0.05 | 0.53 |
|  |  |  |  |  |  |  |  |  |  |
| HOMA-IR | 1274 | 2.8 ± 0.7 (2) | 2.3 ± 1.8 (163) | 3.0 ± 4.0 (1109) | 2.3 ± 3.8 (165) | 2.9 ± 3.9 (1272) | 0.06 | 0.03 | 0.59 |
| HOMA-β | 1274 | 100.4 ± 33.4 (2) | 85.9 ± 77.3 (163) | 100.6 ± 105.6 (1109) | 86.1 ± 102.2 (165) | 98.7 ± 102.5 (1272) | 0.31 | 0.16 | 0.62 |
|  |  |  |  |  |  |  |  |  |  |
| **rs16827243** |  | AA | AG | GG | AA/AG | AG/GG |  |  |  |
|  |  |  |  |  |  |  |  |  |  |
| Fasting p-glucose (mmoL) | 1710 | 5.5 ± 0.4 (4) | 6.1 ± 2.1 (165) | 5.9 ± 1.9 (1541) | 6.0 ± 2.0 (169) | 5.9 ± 2.0 (1706) | 0.51 | 0.32 | NA |
| Fasting insulin (mU/ml) | 1288 | 9.8 (1) | 10.0 ± 9.4 (122) | 10.2 ± 9.1 (1165) | 10.0 ± 9.4 (123) | 10.2 ± 9.2 (1287) | 0.24 | 0.25 | NA |
|  |  |  |  |  |  |  |  |  |  |
| HOMA-IR | 1279 | 2.5 (1) | 3.0 ± 3.5 (121) | 2.9 ± 3.9 (1157) | 3.0 ± 3.5 (122) | 2.9 ± 3.8 (1278) | 0.60 | 0.62 | NA |
| HOMA-β | 1279 | 91.2 (1) | 89.3 ± 83.2 (121) | 99.3 ± 104 (1157) | 89.2 ± 82.9 (122) | 98 ± 102 (1278) | 0.16 | 0.17 | NA |
|  |  |  |  |  |  |  |  |  |  |
| **rs17221756** |  | AA | AG |  |  |  |  |  |  |
|  |  |  |  |  |  |  |  |  |  |
| Fasting p-glucose (mmoL) | 1794 | 5.9 ± 1.9 (1634) | 6.3 ± 2.4 (160) | NA | NA | NA | 0.15 | NA | NA |
| Fasting insulin (mU/ml) | 1323 | 10.2 ± 9.3 (1225) | 10.4 ± 6.2 (98) | NA | NA | NA | 0.34 | NA | NA |
|  |  |  |  |  |  |  |  |  |  |
| HOMA-IR | 1314 | 2.9 ± 3.9 (1216) | 3.0 ± 3.1 (98) | NA | NA | NA | 0.36 | NA | NA |
| HOMA-β | 1314 | 98.4 ± 103.2 (1216) | 102.4 ± 92.8 (98) | NA | NA | NA | 0.59 | NA | NA |
|  |  |  |  |  |  |  |  |  |  |
| **rs9245** |  | AA | AC | CC | AA/AC | AC/CC |  |  |  |
|  |  |  |  |  |  |  |  |  |  |
| Fasting p-glucose (mmoL) | 1771 | 5.5 ± 0.6 (5) | 5.7 ± 1.4 (219) | 5.9 ± 2.0 (1547) | 5.7 ± 1.4 (224) | 5.9 ± 1.9 (1766) | 0.24 | 0.09 | 0.68 |
| Fasting insulin (mU/ml) | 1306 | 14.2 ± 5.9 (3) | 9.1 ± 6.7 (150) | 10.3 ± 9.4 (1153) | 9.2 ± 6.7 (153) | 10.1 ± 9.1 (1303) | 0.15 | 0.24 | 0.17 |
|  |  |  |  |  |  |  |  |  |  |
| HOMA-IR | 1297 | 3.4 ± 1.2 (3) | 2.4 ± 2.0 (150) | 3.0 ± 4.0 (1144) | 2.4 ± 2.0 (153) | 2.9 ± 3.8 (1294) | 0.14 | 0.13 | 0.28 |
| HOMA-β | 1297 | 160 ± 106 (3) | 91 ± 87 (150) | 98 ± 104 (1144) | 92 ± 88 (153) | 97 ± 102 (1294) | 0.23 | 0.48 | 0.15 |
|  |  |  |  |  |  |  |  |  |  |
| **rs4636400** |  | GG | GT | TT | GG/GT | GT/TT |  |  |  |
|  |  |  |  |  |  |  |  |  |  |
| Fasting p-glucose (mmoL) | 1770 | 5.7 ± 1.4 (284) | 6.0 ± 2.1 (826) | 5.9 ± 1.9 (660) | 5.9 ± 2.0 (1110) | 5.9 ± 2.0 (1486) | 0.19 | 0.59 | 0.15 |
| Fasting insulin (mU/ml) | 1308 | 9.9 ± 7.5 (209) | 11 ± 11 (609) | 9.6 ± 7.1 (490) | 10.5 ± 10 (818) | 10.2 ± 9.4 (1099) | 0.62 | 0.36 | 0.98 |
|  |  |  |  |  |  |  |  |  |  |
| HOMA-IR | 1299 | 2.6 ± 2.3 (207) | 3.1 ± 4.7 (605) | 2.7 ± 3.1 (487) | 3.0 ± 4.2 (812) | 3.0 ± 4.0 (1092) | 0.54 | 0.35 | 0.81 |
| HOMA-β | 1299 | 94 ± 91 (207) | 104 ± 118 (605) | 93 ± 82 (487) | 102 ± 112 (812) | 99 ± 103 (1092) | 0.66 | 0.39 | 0.55 |
|  |  |  |  |  |  |  |  |  |  |
| **rs704985** |  | GG | CG | CC | GG/CG | CG/CC |  |  |  |
|  |  |  |  |  |  |  |  |  |  |
| Fasting p-glucose (mmoL) | 1492 | 5.8 ± 1.9 (356) | 6.0 ± 2.2 (709) | 5.8 ± 1.5 (427) | 6.0 ± 2.1 (1065) | 5.9 ± 2.0 (1136) | 0.15 | 0.43 | 0.17 |
| Fasting insulin (mU/ml) | 1093 | 9.7 ± 8.0 (266) | 10.5 ± 10 (510) | 10.2 ± 9.2 (317) | 10.2 ± 9.4 (776) | 10.2 ± 9.3 (827) | 0.65 | 0.46 | 0.77 |
|  |  |  |  |  |  |  |  |  |  |
| HOMA-IR | 1086 | 2.8 ± 3.4 (265) | 3.0 ± 4.0 (506) | 2.9 ± 4.3 (315) | 3.0 ± 4.0 (771) | 2.9 ± 3.8 (821) | 0.45 | 0.35 | 0.65 |
| HOMA-β | 1086 | 100 ± 106 (265) | 100 ± 109 (506) | 95 ± 95 (315) | 99 ± 103 (771) | 100 ± 108 (821) | 0.98 | 0.86 | 0.98 |
|  |  |  |  |  |  |  |  |  |  |

NA = Not Applicable
